# Stiffness and Strength Enhancement of Variable-Thickness Triply Periodic Minimal Surface Shell Lattices via Stress-Feedback-Driven Design

**DOI:** 10.3390/ma19194191

**Published:** 2026-09-30

**Authors:** Zhenfei Li, Winston Wai Shing Ma

**Affiliations:** 1 Research Center of Intelligent Manufacturing Technology, Beijing Institute of Technology, Zhuhai 519088, China; 3220252991@bitzh.edu.cn; 2 Guangdong Engineering Research Center of Intelligent Assembly and Detection Technology, Beijing Institute of Technology, Zhuhai 519088, China; 3 School of Mechanical Engineering, Beijing Institute of Technology, Beijing 100081, China

**Keywords:** TPMS shell lattices, stiffness and strength enhancement, variable thickness design, stress feedback, load-bearing efficiency

## Abstract

Triply periodic minimal surface (TPMS) shell lattices combine high mechanical efficiency with an open-cell topology, yet their stiffness and strength remain well below theoretical upper bounds, leaving considerable room for further enhancement. This study develops a stress-feedback-driven continuous variable-thickness method for simultaneous stiffness and strength enhancement of TPMS shell lattices. Finite-element analyses provide nodal von Mises stresses that are mapped into relaxed thickness updates; spatial filtering and target-volume normalization maintain a smooth thickness field and constant material volume. The method thickens highly loaded regions and thins underutilized regions while keeping the mid-surface geometry unchanged. Across a relative density (RD) range of 5–20% and a maximum-to-minimum thickness-ratio of 100, the effective Young’s modulus and yield strength of N14 shell lattices can be increased by up to 46.39% and 58.82%, respectively. With the thickness ratio restricted to 10, the corresponding maximum enhancementremain 30.90% and 46.75%. The improvement results from transferring material toward existing load paths and increasing the fraction of the shell that participates effectively in load transfer. The proposed method provides a numerically efficient route for designing high-performance lightweight shell lattices. The results obtained with a large thickness-ratio limit represent numerical performance potential and may include locally ultrathin regions, whereas the moderate-contrast thickness fields are manufacturing-oriented numerical candidates that consider the fabrication constraints.

## 1. Introduction

Lightweight lattice materials offer high specific stiffness, specific strength, and energy-absorption potential and have received considerable attention in aerospace, protective engineering, and biomedical applications [1,2]. Conventional truss lattices may develop stress concentrations at discrete joints, and bending-dominated architectures generally exhibit lower stiffness and strength than stretching-dominated plate or shell lattices at the same relative density (RD) [1,3,4,5]. Although stretching-dominated plate lattices can approach theoretical stiffness and strength bounds at moderate RD values, their performance may deteriorate at ultralow RD because of structural buckling [4,5]. Their closed-cell topology can also restrict fluid connectivity, heat and mass transfer, and the removal of residual resin or unmelted powder during additive manufacturing (AM). With recent advances in AM, TPMS shell lattices have emerged as an important class of lightweight structures. They extend periodically along three orthogonal directions and possess smooth, continuous surfaces and interconnected channels, thereby combining mechanical efficiency, open-cell connectivity, and multifunctional potential over a broad range of RD values [6,7,8].

In terms of the development of plate and shell theories, early work by Germain and Lagrange addressed the bending effect of thin flat plates and shallow shells [9]. Subsequently, Kirchhoff–Love thin-shell kinematics describe curved reference surfaces under the assumption that normals to the mid-surface remain normal after deformation, thereby neglecting transverse-shear deformation [9,10,11]. This theory is valid for thin plates and shells where the thickness is much smaller than the characteristic in-plane dimensions. In a curved shell, the initial curvature couples in-plane and transverse displacements. The shell response comprises membrane strain and bending strain associated with changes in curvature, and integrating the corresponding in-plane stresses through the thickness yields membrane-force and bending-moment resultants. For a homogeneous isotropic shell in the linear-elastic regime, the membrane stiffness scales linearly with thickness, whereas bending stiffness scales cubically with the thickness [9,10,11]. In this case, the thickness redistribution may therefore affect these two contributions differently.

Within this shell-mechanics framework, the response of a uniform-thickness TPMS shell lattice is further governed by its underlying topology, RD, curvature distribution, and loading conditions. The material distribution may not necessarily be aligned with the load-transfer paths under a particular load case [12,13,14,15]. Existing studies have expanded the design space through surface offsetting, multiscale hybridization, local reinforcement, and curvature control. Liu et al. investigated surface offsets in Primitive (P), Diamond, Gyroid, and I-WP shell lattices at a constant RD of 15%, in which increasing the second moment of area improved the deformation resistance of some architectures, whereas excessive offsetting weakened the P-type lattices through a plastic-hinge mechanism [16]. Lai et al., Zou et al., and Bian et al. developed hybrid designs that combined TPMS lattices with body-centered-cubic or multiphase TPMS architectures. Their results showed that multiscale or hybrid structures can modify local load transfer, failure progression, and energy absorption, although the outcome depends on the mixing ratio and loading direction [17,18,19]. To address buckling-dominated responses at ultralow RD values, Ding et al. introduced small geometric imperfections to increase the initial bending strain-energy fraction in plate, shell, and truss lattices, thereby delaying buckling and improving stability and strength. This effect depends on RD and lattice type and may become detrimental at moderate to high RD values where material yielding dominates [20]. Ma et al. placed reinforcing ribs along asymptotic and principal-curvature lines, increasing the strength of P- and Neovius (N)-type shell lattices through stress redistribution, suppression of shell buckling, and the incorporation of additional load paths [21]. They subsequently constructed constant-mean-curvature surfaces and achieved a 30.0% increase in the uniaxial strength of P-type lattices at 10% RD, although the zero-mean-curvature TPMS state remained the strongest member of the examined surface family under shear and hydrostatic loading [22]. Additionally, hybrid and hierarchical architectures have likewise been used to improve stiffness, strength, and isotropy simultaneously [23,24,25,26,27]. These findings demonstrate that geometric tailoring can improve load-bearing efficiency for specific architectures and loading conditions, but the benefits remain explicitly dependent on the structural topology, design parameters, and load cases.

In addition to altering the shell mid-surface, variable-thickness design redistributes local material while retaining the underlying topology. Prescribed approaches include the loading-direction thickness gradients of Fan et al. and the three-dimensional grading patterns introduced by Rizza et al.; these designs regulate deformation, failure, and energy absorption, but their thickness fields do not evolve with the stress response [28,29]. Objective-driven approaches provide a larger design space. Ma et al. optimized shell-element thicknesses to obtain elastically isotropic TPMS lattices under an RD constraint [30]. Stress-informed approaches more directly target load transfer: Zhang et al. used a yield-stress threshold to divide a P lattice into reinforced and unreinforced regions [31]; Jia et al. experimentally and numerically demonstrated local-thickness reinforcement of Schwarz P lattices [32]; and Chen et al. used curvature-differentiated Gyroid designs to relieve stress concentration [33]. Zhang et al. subsequently incorporated anisotropic constitutive properties into isotropic variable-thickness shell-lattice design [34], while Liu et al. combined level-set parameterization and thin-shell shape optimization to enhance both stiffness and strength [35]. These studies establish the value of local material redistribution, but threshold partitions, prescribed gradients, and single-field initial designs cannot continuously correct the thickness distribution as the stress field evolves. A continuous stress-to-thickness relation embedded in a repeated analysis-and-update loop is therefore desirable.

Motivated by these studies, this work develops a stress-feedback-driven continuous variable-thickness iteration method for enhancing the stiffness and strength of TPMS shell lattices. The P and N14 mid-surfaces are fixed, nodal thicknesses serve as design variables, and finite-element nodal von Mises stresses drive repeated mapping, filtering, and volume-preserving updates. P and N14 lattices are investigated at RD values of 5%, 10%, 15%, and 20%. Here, *η_t_* = *t*_max_/*t*_min_ denotes the dimensionless maximum-to-minimum nodal-thickness ratio, where *t*_max_ and *t*_min_ are the maximum and minimum nodal shell thicknesses, respectively. Therefore, *η_t_* = 1 represents a uniform-thickness shell, whereas a larger *η_t_* indicates a greater degree of thickness nonuniformity. Three design ranges are considered: a wide numerical design space (*η_t_* ≤ 100) and two moderate-contrast ranges (*η_t_* ≤ 5 and *η_t_* ≤ 10) intended to inform subsequent manufacturing-oriented design. For each thickness-ratio limit, the feasible trajectory state with the largest effective Young’s modulus is selected and its yield strength and stress field are evaluated independently. At 10% RD, the N14 lattice achieves stiffness and yield-strength gains of 46.39% and 50.71%, respectively, in the wide design space; restricting *η*_t_ to 10 retains gains of 30.76% and 38.18%. The improvements arise because the feedback process transfers material away from underutilized regions, reinforces existing load paths, and expands effectively loaded areas. The method therefore offers a simple, load-dependent route for improving material utilization without increasing mass and provides a quantitative basis for selecting thickness contrast for subsequent manufacturing-oriented design. As a comparison, conventional inverse analysis typically minimizes the discrepancy between experimental measurements and forward simulations to identify residual stresses or constitutive parameters [36,37]. Unlike these approaches, the present method does not infer unknown physical states from experimental data, strictly distinguishing it from inverse identification. It does, however, employ a conceptually similar analysis-update loop, wherein finite-element nodal stresses are used to iteratively update a volume-preserving thickness field. Consequently, this approach is best characterized as a stress-informed design framework—one that could readily be coupled with experimentally driven inverse identification in future applications.

## 2. Methods

### 2.1. Geometric Modelling and Finite-Element Analysis

**Figure 1:**
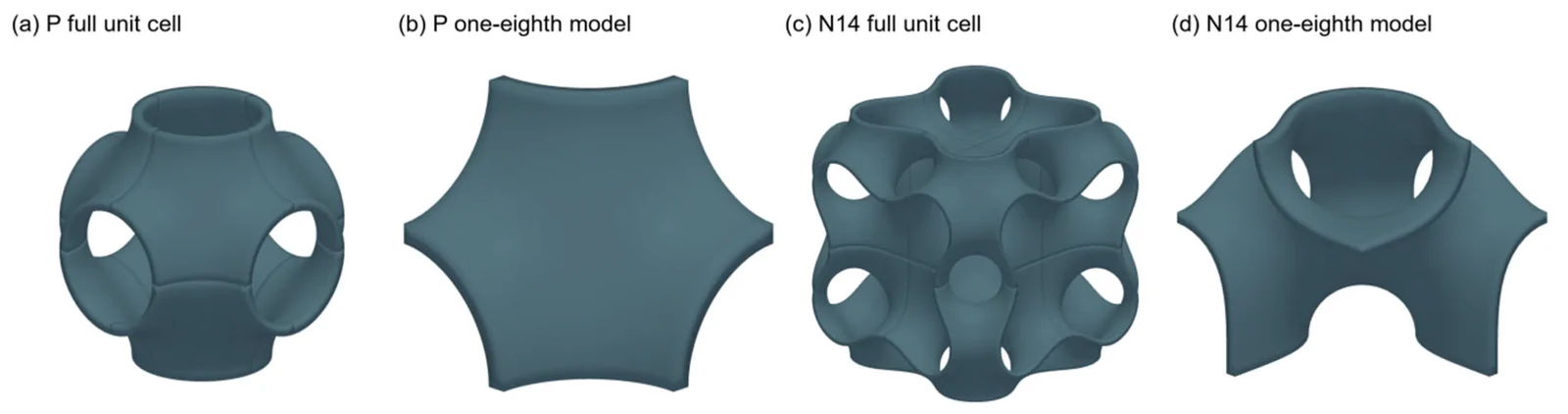
Geometric models used in the finite-element analyses: (**a**) reconstructed P full unit cell; (**b**) P one-eighth model; (**c**) reconstructed N14 full unit cell; and (**d**) N14 one-eighth model. The full cells are obtained by reflection of the corresponding analysis models about the three symmetry midplanes.

Schwarz P and N14 TPMS shell lattices are selected as the study objects. High-accuracy triangulated mid-surfaces are generated and relaxed using Surface Evolver following established TPMS shell-lattice modelling procedures [30,38]. Reflection about the three symmetry midplanes reconstructs the full periodic unit cell from the one-eighth analysis model, as shown in Figure 1. Both architectures use the same periodic length scale and discretization procedure; only nodal thickness varies, while the mid-surface geometry and mesh connectivity remain fixed. Each one-eighth model is normalized to a cube of edge length *L* = 1 mm. The P model contains 865 nodes and 1632 triangular elements, whereas the N14 model contains 2216 nodes and 4236 triangular elements.

The RD $\bar{\rho }$ is defined as the ratio of material volume *V*_s_ to the unit-cell envelope volume *V*_c_ = *L*^3^, and is prescribed at 5%, 10%, 15%, and 20% in this study. The corresponding target material volume is *V*_tar_ = $\bar{\rho }$ *V*_c_. Under the thin-shell volume approximation used by the thickness-update procedure,

(1) $$\bar{\rho }=\frac{V_{s}}{V_{c}},V_{s}\approx \sum_{e=1}^{N_{e}}A_{e}{\bar{t}}_{e},{\bar{t}}_{e}=\frac{t_{e1}+t_{e2}+t_{e3}}{3}.$$

where *A*_e_ and ${\bar{t}}_{e}$ are the mid-surface area and mean nodal thickness of triangular element *e*, respectively. The mid-surface area approximation in Equation (1) is employed solely to evaluate the volume of shell lattices. It is not used to approximate the stress, strain, or stiffness response of the shell, which is obtained from the finite-element shell formulation. The initial thickness of each uniform-thickness lattice is determined from the target RD, envelope volume, and total mid-surface area. Because the input file stores thickness values with a finite number of decimal places, slight rounding errors are unavoidable; the volume constraint is therefore referenced to *V*_tar_ rather than to the rounded baseline volume. Here, “constant RD” means that, for the same mid-surface discretization and the thin-shell approximation in Equation (1), the material volume is normalized to *V*_tar_ after every iterative update. The mid-surface geometry, nodal coordinates, and element connectivity remain fixed throughout the iteration, so the thickness redistribution is the only difference among the thickness states of a given architecture. Here, *N_e_* denotes the total number of triangular shell elements.

The finite-element models employ the Abaqus(v2024) three-node linear triangular general-purpose shell element S3, with spatially varying thickness specified through nodal thicknesses. According to the Abaqus formulation, the S3 shell element is a three-node general-purpose conventional shell element derived from S4R. It accounts for membrane deformation, changes in curvature, and transverse-shear deformation, and permits finite membrane strains and large rotations. As the shell becomes thin, transverse-shear deformation diminishes and the element approaches discrete Kirchhoff-type thin-shell behavior. Five section points based on Simpson integration were specified through the shell thickness to evaluate the constitutive response. Because the present analyses are static, neither translational nor rotary inertia affects the numerically evaluated mechanical responses. Therefore, although the nodal geometry represents the shell reference mid-surface, the finite-element model is not a membrane-only formulation and retains the bending stiffness and through-thickness integration of the shell. The shell discretization and symmetry-reduced analysis follow the variable-thickness TPMS modelling approach reported by Ma et al. [30]. The parent material is represented by an isotropic elastoplastic constitutive model with *E*_s_ = 189,310 MPa and *ν*_s_ = 0.3. Plasticity is defined by a true-stress–plastic-strain table whose first entry is 520.268 MPa at zero plastic strain. A Static, General step is used with geometric nonlinearity enabled; comparable elastoplastic descriptions and geometrically nonlinear analyses have been applied to additively manufactured stainless steel 316L shell lattices [39].

The boundary conditions combine periodic displacement relations with reflection symmetry about the three midplanes of the one-eighth TPMS models [30,40]. The corresponding normal displacements and rotations are constrained on the symmetry planes, and each positive boundary face is kinematically coupled to its associated reference point. This arrangement preserves displacement compatibility between adjacent periodic cells. An axial displacement of magnitude 0.01*L* is prescribed at the reference point in the target direction, corresponding to a nominal axial strain of 1% at the end of loading. The reference points in the other two directions retain their normal translational degrees of freedom, allowing transverse contraction or expansion while satisfying the periodic relations. From the reference-point displacement *u* and reaction force *F*, nominal strain and stress are calculated as *ε* = |*u*|/*L* and *σ* = |*F*|/*L*^2^, respectively; absolute values report the compressive response as positive. Outputs include reference-point displacements and reaction forces together with shell-element stress, strain, and equivalent plastic strain.

### 2.2. Stress-Feedback-Based Continuous Variable-Thickness Design

**Figure 2:**
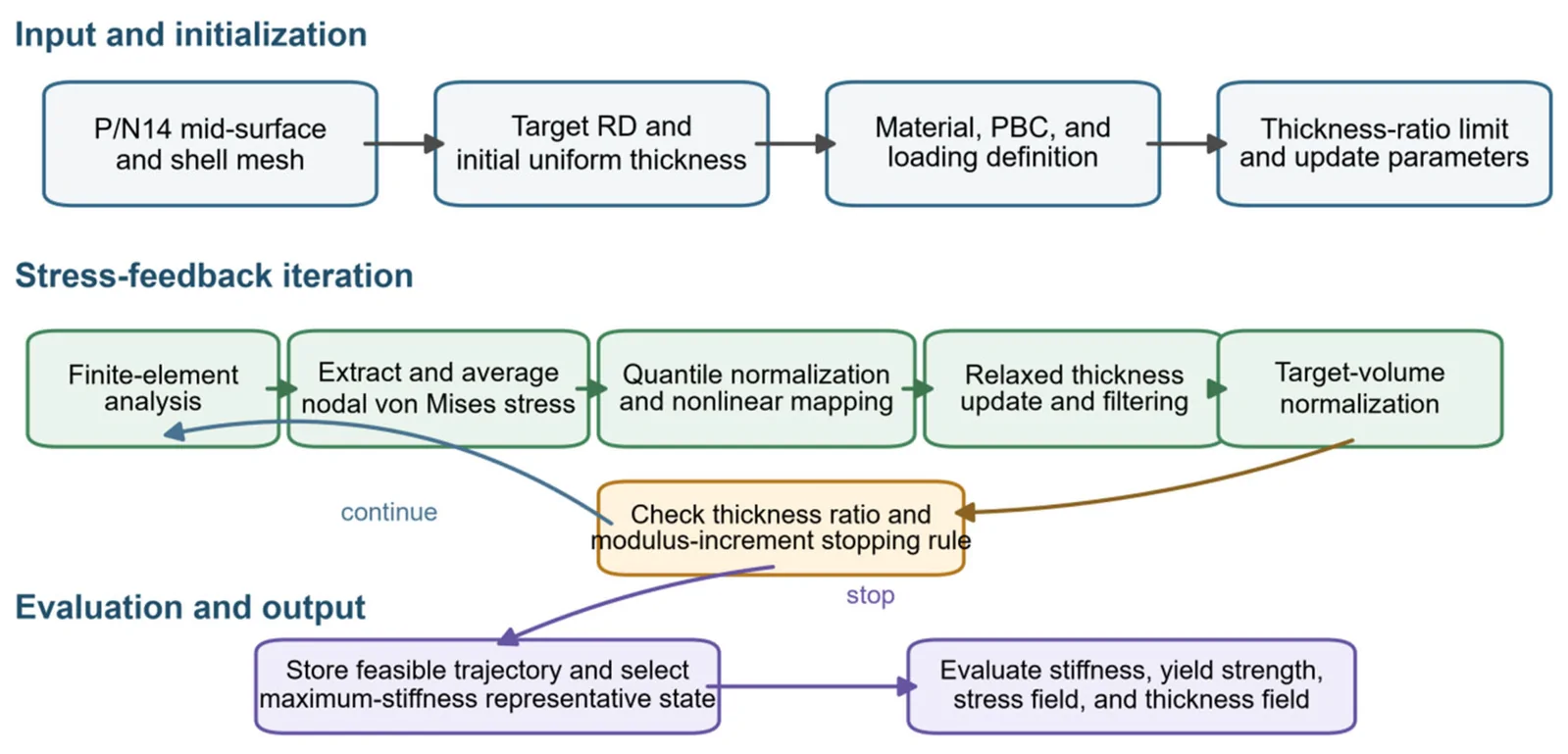
Overall workflow of the stress-feedback-driven continuous variable-thickness method, showing initialization, the closed finite-element analysis and thickness-update loop, the quantified stopping check, and representative-state evaluation.

The complete stress-feedback loop is summarized in Figure 2. Let *s*_i_^(n)^ and *t*_i_^(n)^ denote the nodal von Mises equivalent stress and nodal thickness, respectively, at iteration *n*. The stress-feedback variable is the nodal von Mises stress evaluated at the third through-thickness section point (SP3), located at the shell reference mid-surface, in the final frame corresponding to 1% nominal axial strain. The requested field output is averaged at the nodes; because a geometric node may receive multiple stress records from adjacent shell elements, records with the same node label are further grouped and arithmetically averaged before being paired one-to-one with the nodal thickness field. The resulting scalar stress field is used as an indicator of the mid-surface load-transfer pattern and is normalized using its 10th and 90th percentiles:

(2) $$x_{i}^{\left(n\right)}=\mathrm{clip}\left(\frac{s_{i}^{\left(n\right)}-s_{10}^{\left(n\right)}}{s_{90}^{\left(n\right)}-s_{10}^{\left(n\right)}},0,1\right).$$

where *x*_i_^(n)^ is the normalized stress indicator at node i and iteration n, and clip(*y*, 0, 1) = min[max(*y*, 0), 1] truncates values below 0 and above 1 to 0 and 1, respectively. If *s*_90_^(n)^ = *s*_10_^(n)^, *x*_i_^(n)^ is set to 0.5. Using the 10th and 90th percentiles rather than the extrema reduces the influence of a few anomalously high-stress or low-stress nodes, while truncation assigns bounded update weights outside the percentile interval. A sigmoid mapping and relaxed update are then applied:

(3) $$g_{i}^{\left(n\right)}=\frac{1}{1+\exp\left[-6\left(x_{i}^{\left(n\right)}-0.5\right)\right]},\;f_{i}^{\left(n\right)}=0.90+0.20g_{i}^{\left(n\right)},\;t_{i,r}^{\left(n+1\right)}=(1-\alpha )t_{i}^{\left(n\right)}+\alpha f_{i}^{\left(n\right)}t_{i}^{\left(n\right)},\alpha =0.80.$$

Here, *g_i_*^(^*^n^*^)^ is the sigmoid-mapped stress factor, *f_i_*^(^*^n^*^)^ is the bounded thickness multiplier, *t_i,r_*^(^*^n^*^+1)^ is the relaxed pre-filter thickness, and α is the relaxation coefficient. This mapping preserves the relative ordering of the normalized stresses, assigning larger thickness-update weights to relatively highly stressed nodes. The relaxation coefficient controls the degree of mixing between the old and new thickness fields. Because the mapping factor is bounded between 0.90 and 1.10, it governs only the relative update within one iteration and does not impose absolute manufacturing limits on thickness.

A distance-weighted spatial filter is applied after the relaxed update. The neighborhood of node *i* is *N*_i_ = {*j* | *d*_ij_ ≤ *R*}, where *d*_ij_ is the Euclidean distance between nodes *i* and *j* and *w*_ij_ = 1 − *d*_ij_/*R*. Filter radii *R* = 0.0705*L* and 0.0600*L* are used for the P and N14 lattices, respectively, so that the neighborhood spans at least the local adjacent-node distance. Such filters suppress local oscillations and mesh dependence in discrete design variables [41,42]. The filtered thickness is

(4) $${\tilde{t}}_{i}^{\left(n+1\right)}=\frac{\sum_{j\in N_{i}}w_{\mathrm{ij}}t_{j,r}^{\left(n+1\right)}}{\sum_{j\in N_{i}}w_{\mathrm{ij}}}.$$

Finally, the filtered material volume ${\tilde{V}}_{s}^{\left(n+1\right)}$ is calculated, and all nodal thicknesses are scaled uniformly:

(5) $$t_{i}^{\left(n+1\right)}={\tilde{t}}_{i}^{\left(n+1\right)}\frac{V_{\mathrm{tar}}}{{\tilde{V}}_{s}^{\left(n+1\right)}}.$$

Spatial filtering suppresses thickness oscillations between neighboring nodes, whereas volume scaling restores the material volume to the target value associated with the prescribed RD; the two operations serve different purposes and are applied sequentially. The normalized thickness field is returned to the finite-element model, and the updated stress field drives the next correction, forming a closed analysis–update–analysis loop. Because filtering and volume scaling jointly affect nodal thickness, the final absolute thickening or thinning of a node cannot be inferred from *f*_i_^(n)^ alone. The update uses only the nodal stress at the prescribed loading endpoint; Young’s modulus, yield strength, and stress dispersion are not included in the update equation. If an updated model fails to converge, the automated procedure progressively blends the candidate thickness field back toward the most recent converged field and reapplies target-volume normalization after each rollback. If every candidate fails, the last converged state is retained so that a single unsuccessful analysis does not invalidate the complete iteration trajectory.

### 2.3. Iterative Optimization and Performance Evaluation

The initial models have uniform thickness, and the stress-feedback iterations progressively generate nonuniform nodal-thickness fields. Thickness contrast is quantified by *η*_t_ = *t*_max_/*t*_min_. Three nested numerical ranges are considered: *η*_t_ ≤ 5 and *η*_t_ ≤ 10 impose moderate thickness contrast to support subsequent manufacturing-oriented design, whereas *η*_t_ ≤ 100 explores the performance potential available in a wider numerical space. These bounds constrain only relative thickness contrast; they do not impose an absolute minimum wall thickness, build-orientation restriction, curvature-dependent dimensional tolerance, or post-processing-accessibility constraint.

Automated iterations are performed for the P and N14 lattices at all four RD values. The uniform-thickness baseline and every successfully solved thickness field and mechanical response are retained. Let *I*_B_ = {*n* | *η*_t_^(n)^ ≤ *B*} denote the feasible trajectory states under thickness-ratio limit *B*, where *B* ∈ {5, 10, 100}. The representative state is defined as

(6) $$n_{B}^{*}=\underset{n\in I_{B}}{\mathrm{argmax}}E_{n}^{\mathrm{eff}}.$$

In Equation (6), *E_n_^eff^* denotes the effective Young’s modulus at iteration *n*. Here, *n*_B_* is the iteration index of the representative state within *I*_B_, selected by the maximum effective Young’s modulus in the target loading direction. Yield strength and stress distribution are supplementary descriptors and do not participate in state selection; thus, the procedure is not a multi-objective optimization. Results for *η*_t_ ≤ 100 are trajectory maxima rather than proof of global optimality over all admissible thickness fields. For automated control, the Young’s-modulus estimate evaluated during iteration *n* is denoted *E*_iter_^(n)^, and the relative increment between consecutive iterations is

(7) $$g_{E}^{\left(n\right)}=\frac{E_{\mathrm{iter}}^{\left(n\right)}-E_{\mathrm{iter}}^{\left(n-1\right)}}{\max\left(\left|E_{\mathrm{iter}}^{\left(n-1\right)}\right|,10^{-15}\right)}.$$

Comparison begins at iteration 1. The automated trajectory stops immediately after the first non-positive increment, gE(n) ≤ 0, meaning that Eiter(n) does not exceed Eiter(n − 1); no additional numerical tolerance or consecutive-iteration window is used. A hard cap of 100 iterations prevents indefinite continuation if all increments remain positive. This rule controls only automated solution generation. For consistent reporting, *E*^eff^ is subsequently recalculated for every saved state using the unified linear-regression procedure below, and the representative state under each thickness-ratio limit is reselected from these refitted values.

For a given architecture, mesh, RD, and nominal strain of 1%, the mean *μ*_s_, standard deviation *σ*_s_, and coefficient of variation *CV*_s_ = *σ*_s_/*μ*_s_ of the valid nodal-stress set (containing *N* nodes) are used to characterize the stress distribution. The 90th and 95th percentiles, *s*_90_ and *s*_95_, are also reported to describe the high-stress tail, whereas the maximum stress is used only as an auxiliary indicator. These statistics are compared at the same nominal strain rather than at the same macroscopic stress and must therefore be interpreted together with the reference-point reaction force. A smaller *CV*_s_ indicates lower relative dispersion of nodal stress but does not, by itself, imply improved load-bearing performance. Thickness states are compared only within the same architecture, mesh, and RD; comparisons of nodal statistics between the P and N14 lattices must also account for their different mesh discretizations. In addition to *η*_t_, the thickness distribution is characterized by *t*_90_/*t*_10_ and *CV*_t_ = *σ*_t_/*μ*_t_, where *μ*_t_ and *σ*_t_ are the mean and standard deviation of nodal thickness. These supplementary metrics reduce the sensitivity of thickness-nonuniformity assessment to a few extreme nodes.

The effective Young’s modulus in the target direction is obtained by least-squares fitting with an intercept to all output points within *ε* ≤ 0.001. The coefficient of determination *R*^2^ is reported to verify the linearity of the initial response. The 0.2% offset line is obtained by shifting the fitted elastic line by 0.002 along the positive strain axis:

(8) *σ* = *E*^eff^*ε* + *b*,*σ*_off_ = *E*^eff^(*ε* − 0.002) + *b*.

where *b* is the fitted intercept. The offset line has the same slope as the elastic fit but a stress-axis intercept of *b* − 0.002*E*^eff^. The first intersection between the offset line and the nominal stress–strain curve defines the 0.2% offset effective yield point; linear interpolation is applied between adjacent discrete points. The RD-normalized performance indices are defined as

(9) $${\overset{―}{E}}_{\mathrm{sp}}=\frac{E^{\mathrm{eff}}}{\bar{\rho }E_{s}},{\overset{―}{\sigma }}_{y,\mathrm{sp}}=\frac{\sigma_{y}^{\mathrm{eff}}}{\bar{\rho }\sigma_{\mathrm{ys}}},\sigma_{\mathrm{ys}}=520.268\;\mathrm{MPa}.$$

The ratios *E*^eff^/*E*_s_ and *σ*_y_^eff^/*σ*_ys_ and their percentage changes relative to the corresponding uniform-thickness baseline are also reported. Satisfaction of the constant-RD constraint is quantified by the numerical volume error *δ*_V_ = (*V*_s_ − *V*_tar_)/*V*_tar_ × 100%. The final results include the actual thickness ratio, iteration number, thickness percentiles, stress statistics, effective properties, and numerical volume error. These quantities are used to relate material redistribution, stress distribution, and target-direction performance. Except for *E*^eff^, which is used to select representative states along a trajectory, none enters the thickness-update rule, and no monotonic increase in performance with thickness ratio is assumed.

### 2.4. Parameter and Mesh-Sensitivity Analysis

To evaluate the numerical robustness of the proposed method, we assessed both parameter and mesh sensitivity at a relative density (RD) of 10%. For the algorithmic parameters, a one-factor-at-a-time analysis was performed on both the P and N14 topologies. The baseline configuration used a relaxation coefficient of α = 0.80, a sigmoid-based thickness-multiplier range of [0.90, 1.10], and spatial filter radii of 0.0705L (for P) and 0.0600L (for N14). From this baseline, α was varied between 0.60 and 1.00, the multiplier ranges were adjusted to [0.95, 1.05] and [0.85, 1.15], and the filter radii were scaled by factors of 0.8 and 1.2.

Additionally, relative online-modulus-growth thresholds of 0, 0.0001, and 0.0005 were evaluated retrospectively using saved design trajectories. All other settings—including geometry, material properties, loading, volume constraints, and post-processing—were held constant. For each trajectory, the representative design was selected as the state exhibiting the highest effective Young’s modulus while satisfying the thickness-ratio constraint (*η_t_* ≤ 10). It should be noted that this assessment aims to verify robustness within specific parameter intervals, rather than to identify a parameter-independent global optimum.

Mesh sensitivity was evaluated separately for the P topology at 10% RD by comparing the baseline mesh (1632 S3 elements) with two independent meshes (1200 and 6528 elements). The maximum variation in mid-surface area across these three meshes was a negligible 0.041%. To maintain a consistent material-volume constraint, the uniform thickness of each mesh was individually scaled to achieve an exact RD of 10%, keeping all other settings unchanged. A minor 0.058% difference in modulus was observed between the exact-volume coarse mesh (uniform thickness of 0.0852399*L*) and the Table 1 baseline (thickness of 0.0852*L*). However, this discrepancy stemmed entirely from volume rounding rather than actual mesh effects. Finally, the effective Young’s modulus, 0.2% offset yield strength, and nominal stress at 1% strain were compared. Because these meshes were generated via independent tessellations rather than systematic nested refinement, this analysis serves as a practical evaluation of the global mechanical response rather than a formal asymptotic mesh-convergence study.

## 3. Results and Discussion

### 3.1. Iterative Evolution and Performance Potential in the Wide Design Space

**Figure 3:**
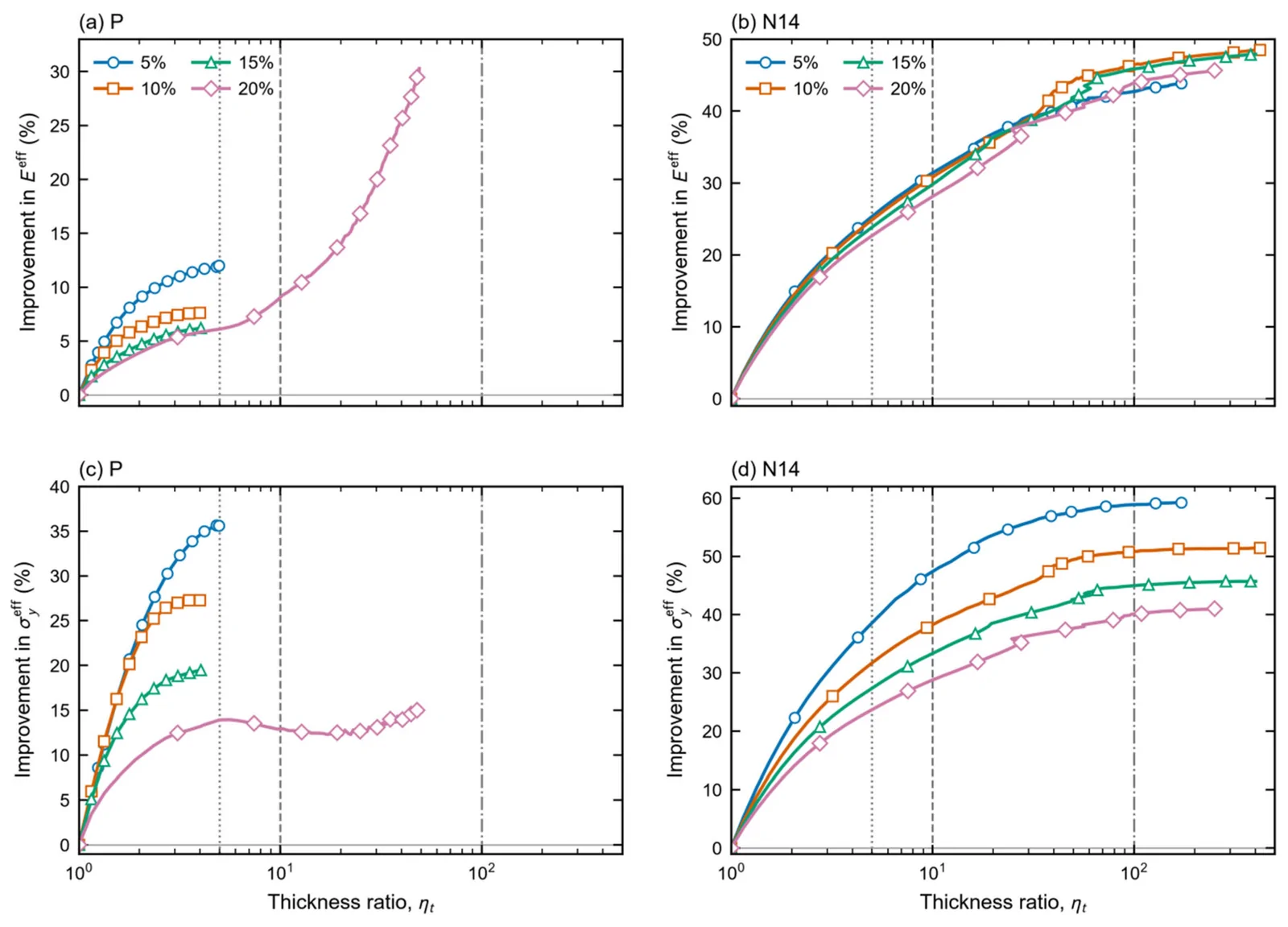
Stiffness and yield-strength evolution along the stress-feedback trajectories: (**a**,**c**) P and (**b**,**d**) N14 shell lattices at RD values of 5%, 10%, 15%, and 20%. The upper panels show effective Young’s-modulus improvement and the lower panels show yield-strength improvement relative to the corresponding uniform-thickness baseline. Vertical lines denote *η*_t_ = 5, 10, and 100.

To evaluate performance potential under weak thickness restrictions, the complete feasible trajectories within *η*_t_ ≤ 100 are examined. Figure 3 presents both the effective Young’s-modulus and yield-strength changes relative to each uniform-thickness baseline. Every stiffness value is refitted from the corresponding stress–strain response using *ε* ≤ 0.001, while yield strength is obtained from the 0.2% offset construction. The coefficients of determination for all eight baselines and representative states exceed 0.999998, confirming stable initial-stiffness estimation. During the early iterations, all models show positive stiffness and yield-strength gains as material moves from relatively low-stress regions toward load-bearing regions; the later evolution depends strongly on topology and RD.

The two topologies exhibit distinct trajectories. The P models at RD values of 5%, 10%, and 15% reach their maximum computed stiffness at *η*_t_ ≈ 4–5, with gains of 12.00%, 7.63%, and 6.20%, respectively, after which the first non-positive iteration-level modulus increment terminates each trajectory. Their yield-strength gains at the selected states are 35.64%, 27.27%, and 19.48%. The 20% P model continues to benefit from larger thickness contrast and reaches a 30.28% stiffness gain and a 15.15% yield-strength gain at *n* = 95 and *η*_t_ = 49.05. Thus, stiffness and yield strength need not evolve identically because representative states are selected using stiffness alone.

By comparison, all four N14 curves maintain a relatively steady upward trend over a much wider range of *η*_t_. After representative states are selected according to Equation (6) within *η*_t_ ≤ 100, the effective Young’s moduli of the 5%, 10%, 15%, and 20% models increase by 42.55%, 46.39%, 45.85%, and 43.81%, respectively, and their actual thickness ratios range from 91.37 to 97.58 (Table 1). This result indicates that the N14 topology can continue to exploit increasing thickness contrast over a broad range of continuous material redistribution and that its improvement is less sensitive to RD than that of the P topology. This distinction reflects the response of the two topologies for the present loading direction and computed feedback trajectories and should not be interpreted as general superiority of N14 under other loading conditions or design objectives.

Under the thin-shell volume calculation used by the algorithm, the volume deviations of all eight representative states from their target values are on the order of 10^−12^%; consequently, the stiffness changes are not caused by an increase in total material volume. The performance gains in the wide design space are, however, accompanied by locally ultrathin regions. The minimum nodal thicknesses of the N14 representative states range from 5.41 × 10^−4^*L* to 1.91 × 10^−3^*L*, producing approximately two orders of magnitude of thickness contrast. These states are useful for assessing the numerical performance potential of the algorithm, but their local dimensions may not satisfy practical additive-manufacturing requirements. The wide-design-space results should therefore be regarded as a performance reference for subsequent constrained designs rather than as directly manufacturable solutions. The next section examines the improvements retained under *η*_t_ ≤ 5 and *η*_t_ ≤ 10.

**Figure 4:**
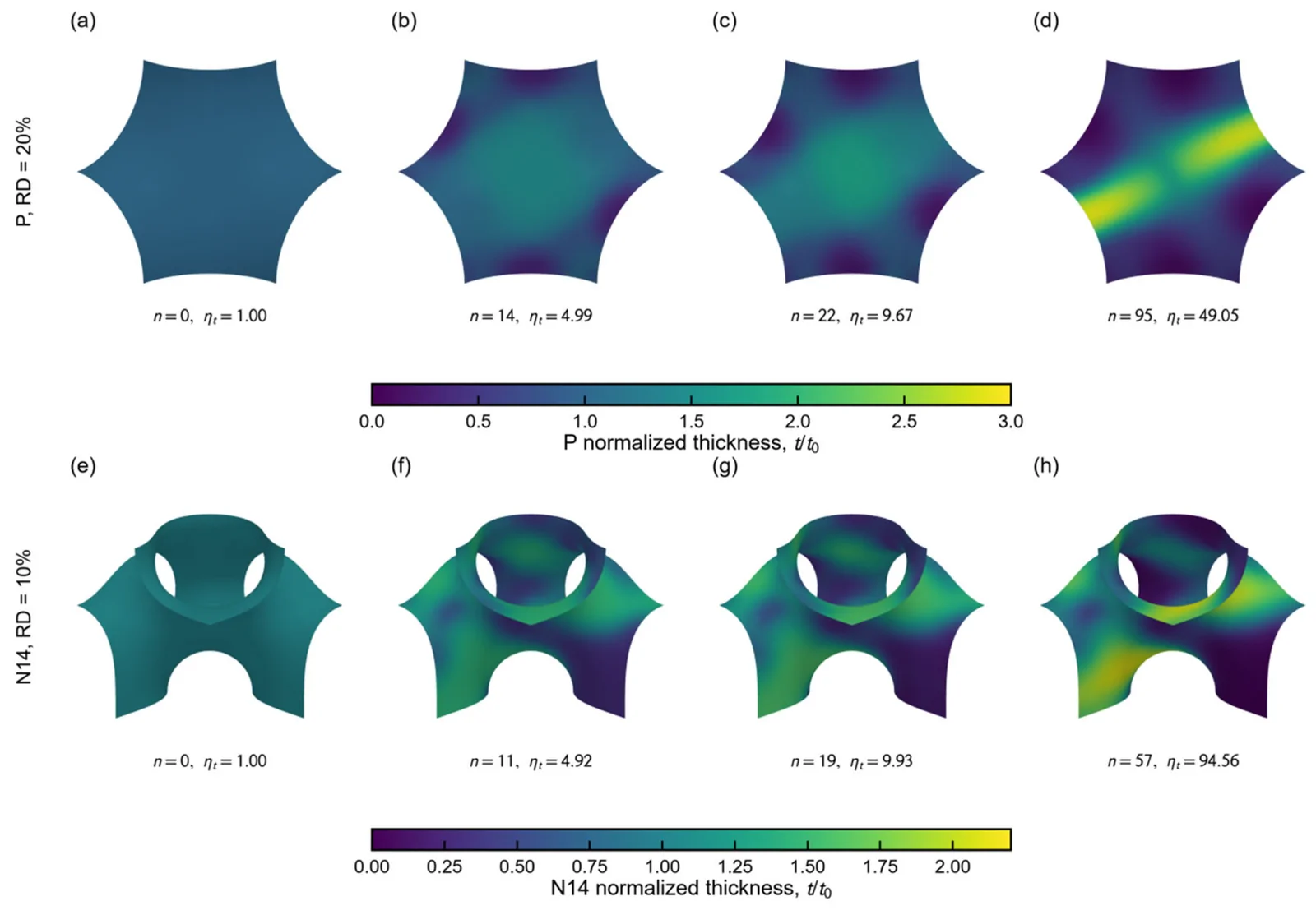
Evolution of normalized thickness fields along representative trajectories: (**a**–**d**) P lattice at 20% RD for *n* = 0, 14, 22, and 95, corresponding to *η*_t_ = 1.00, 4.99, 9.67, and 49.05; and (**e**–**h**) N14 lattice at 10% RD for *n* = 0, 11, 19, and 57, corresponding to *η*_t_ = 1.00, 4.92, 9.93, and 94.56. Within each row, colors are mapped onto a fixed undeformed mid-surface and view; separate normalized-thickness scales are used.

Figure 4 connects the macroscopic trajectories to the evolving thickness fields. The 20% RD P model is shown because it is the only P case that continues into the wide design space: the *η*_t_ ≤ 5, *η*_t_ ≤ 10, and wide-space states provide stiffness gains of 6.11%, 8.79%, and 30.28% and yield-strength gains of 13.91%, 12.88%, and 15.15%, respectively. For the 10% RD N14 model, the corresponding states provide stiffness gains of 24.71%, 30.76%, and 46.39% and yield-strength gains of 31.49%, 38.18%, and 50.71%. In both topologies, thickened and thinned regions grow along continuous surface bands rather than isolated elements. Moderate contrast already establishes the main load-bearing partition, whereas the wider design space amplifies the same pattern and introduces locally ultrathin regions.

### 3.2. Performance Retention Under Moderate Thickness-Contrast Constraints

To establish a direct connection between performance potential and restricted thickness contrast, the upper thickness-ratio limit is reduced to *η*_t_ ≤ 5 and *η*_t_ ≤ 10. For every topology and RD, the state with the maximum *E*^eff^ among the computed states satisfying the corresponding limit is selected according to Equation (6), without retuning the update parameters. Both constrained results therefore originate from the same stress-feedback trajectory, allowing their performance differences to be attributed to the available thickness-contrast range. Because selection is based solely on stiffness, the subsequently calculated 0.2% offset yield strength is an independent evaluation metric and does not affect state selection.

**Figure 5:**
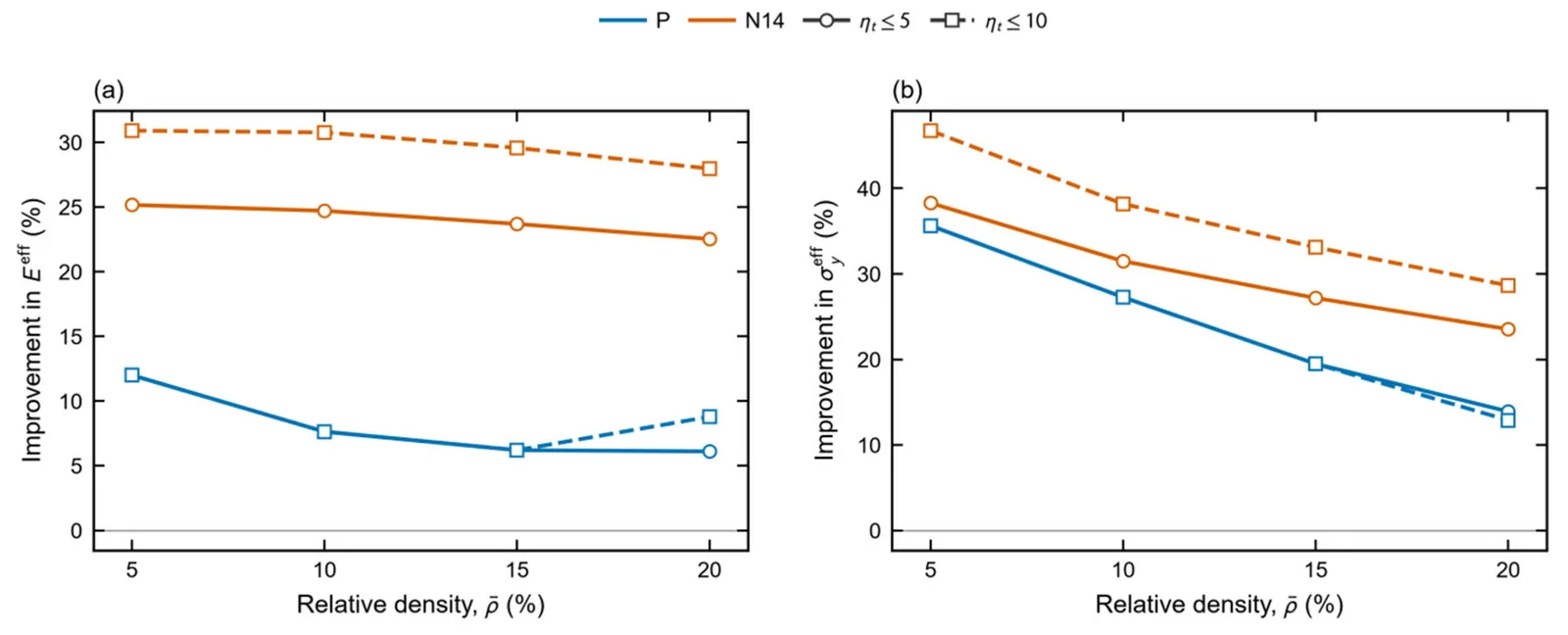
Performance improvements under moderate thickness-contrast constraints: (**a**) effective Young’s modulus and (**b**) yield strength. Colors distinguish topology, while line styles and markers distinguish the thickness-ratio limit.

Figure 5a shows that the N14 topology provides consistent stiffness gains under both constraints. For *η*_t_ ≤ 5, *E*^eff^ increases by 22.52–25.16% across the four RD values, with a mean increase of 24.02%. Relaxing the limit to 10 increases the range to 27.96–30.90%, with a mean of 29.80%. Relative to the 42.55–46.39% improvements in the wide design space, the *η*_t_ ≤ 5 and *η*_t_ ≤ 10 states retain approximately 51–59% and 64–73% of the computed stiffness gains, respectively. Thus, most of the early benefit of the N14 topology does not depend on an extreme, nearly 100-fold thickness contrast; a moderate contrast is sufficient to establish nonuniform load paths with substantial gains.

The constrained P results exhibit stronger dependence on RD. At RD values of 5%, 10%, and 15%, the computed trajectories reach their maximum stiffnesses at *η*_t_ = 4.97, 4.00, and 4.04, respectively. Increasing the admissible limit from 5 to 10 therefore does not change the representative states, and the corresponding stiffness improvements remain 12.00%, 7.63%, and 6.20%. The 20% model improves by 6.11% under *η*_t_ ≤ 5 and by 8.79% under *η*_t_ ≤ 10, still well below the 30.28% gain found in the wide design space. Additional thickness contrast can therefore release considerable stiffness potential in the high-RD P model, whereas the benefit of the present feedback trajectory saturates earlier at lower RD values and is not increased merely by relaxing the upper thickness-ratio limit.

The yield metric follows a trend that is broadly consistent with, but not identical to, that of stiffness (Figure 5b and Table 2). Under *η*_t_ ≤ 5 and *η*_t_ ≤ 10, the yield strength of the N14 lattice increases by 23.53–38.29% and 28.64–46.75%, respectively, indicating that constrained material redistribution not only increases initial stiffness but also delays yielding in critical regions. For the four P models, the yield improvement under *η*_t_ ≤ 5 ranges from 13.91% to 35.64%, with the low-RD models showing substantially larger yield strength enhancement than stiffness improvement. When the limit for the 20% P model is relaxed from 5 to 10, its stiffness gain increases from 6.11% to 8.79%, whereas its yield gain decreases slightly from 13.91% to 12.88%. This is not a numerical inconsistency but a trade-off revealed by stiffness-based state selection: a thickness distribution that improves the linear-elastic load path is not necessarily optimal for controlling stress at the location of initial yielding.

From a geometric perspective, the minimum nodal thicknesses among the eight representative models are no smaller than 7.13 × 10^−3^*L* for *η*_t_ ≤ 5 and 4.15 × 10^−3^*L* for *η*_t_ ≤ 10. These values are approximately 13.2 and 7.7 times the global minimum of 5.41 × 10^−4^*L* in the wide design space. The thickness-ratio constraints thus strongly suppress ultrathin regions while retaining appreciable stiffness and yield gains. Nevertheless, *η*_t_ describes only relative thickness dispersion and cannot independently establish manufacturability. The absolute minimum wall thickness still depends on unit-cell size *L*, material system, fabrication process, and post-processing capability. The present results therefore define numerical design windows for manufacturing-oriented development, for which the admissible thickness contrast should be selected jointly with unit-cell size, printing resolution, material system, and application-specific dimensional requirements. Recent STL-free hybrid toolpath generation for laser-based powder bed fusion directly translates implicit shell geometries into laser paths and has enabled ultrathin-walled microscale shell lattices with improved geometric fidelity, illustrating that attainable wall thickness depends strongly on the selected process and toolpath strategy [43].

### 3.3. Mechanisms of Thickness Redistribution and Stress Regulation

The preceding sections demonstrate that nonuniform thickness can improve effective properties, but macroscopic metrics alone do not reveal the source of the gains. To examine how the feedback process modifies local load transfer, the nodal thickness and nodal von Mises stress of the representative states under *η*_t_ ≤ 5 and *η*_t_ ≤ 10 are analyzed. In addition to the thickness coefficient of variation *CV*_t_ and stress coefficient of variation *CV*_s_ defined in Section 2.3, the Pearson correlation coefficient *r*_t,s0_ is used to quantify the linear relationship between the representative-state thickness *t* and the initial stress *s*_i_^(0)^ at the same node. This correlation is not used by the optimization; it only indicates whether the final material distribution retains information about the initial load path.

**Figure 6:**
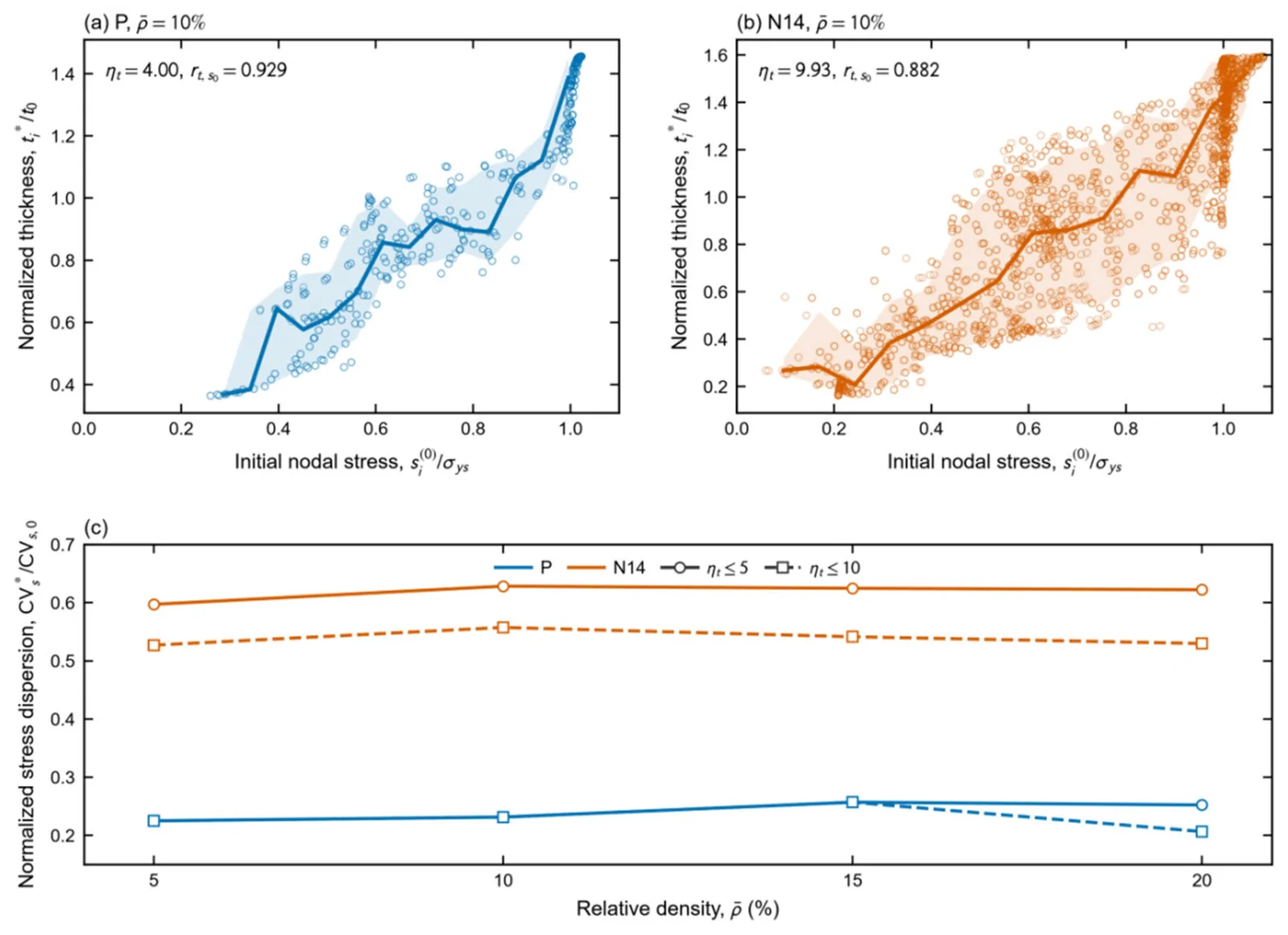
Thickness redistribution and nodal-stress regulation: relationships between normalized nodal thickness and initial nodal stress for the representative states under *η*_t_ ≤ 10 in the 10% (**a**) P and (**b**) N14 models; solid lines and shaded bands indicate the binned medians and the 10th–90th percentile intervals, respectively. (**c**) Stress dispersion normalized by the corresponding uniform-thickness baseline under the two moderate thickness-ratio constraints.

Figure 6a,b show the relationship between nodal thickness and initial stress for the representative states under *η*_t_ ≤ 10 at 10% RD. The P state has *η*_t_ = 4.00 and *r*_t,s0_ = 0.929, while the N14 state has *η*_t_ = 9.93 and *r*_t,s0_ = 0.882. Both show relatively strong positive correlations: nodes carrying high stress initially generally receive greater relative thickness, whereas low-stress nodes are thinned. The finite scatter arises because the final thickness depends on relaxation, neighborhood filtering, volume normalization, and repeated stress redistribution rather than a one-step linear rescaling of the initial stress field.

After material is transferred toward highly stressed regions, the nodal-stress distribution narrows markedly. In Figure 6c, *CV*_s_/*CV*_s,0_ denotes the stress dispersion of a representative state normalized by that of its own uniform-thickness baseline. For the P topology, this ratio is 0.225–0.257 under *η*_t_ ≤ 5, corresponding to a 74.31–77.51% reduction in *CV*_s_, and decreases further to 0.206–0.257 under *η*_t_ ≤ 10, corresponding to a reduction of 74.31–79.36%. The normalized dispersion of the N14 topology is higher than that of the P topology but still decreases from 0.597–0.628 under *η*_t_ ≤ 5 to 0.527–0.557 under *η*_t_ ≤ 10, representing reductions of 37.17–40.30% and 44.27–47.31%, respectively. The narrow ranges across the four RD values show that the reduction in stress dispersion is a robust outcome of the feedback update rather than an anomaly at a single density.

The reduction in stress dispersion is not primarily caused by a large decrease in the high-stress tail. As shown in Table 3, the 95th percentile stress *s*_95_ of every moderately constrained representative state changes by only −1.26% to +0.05% relative to its baseline. At the same nominal strain of 1%, meanwhile, the mean nodal stress increases by approximately 10.9–31.8%. The feedback design therefore acts mainly by thinning underutilized regions and reinforcing pre-existing high-stress load paths, thereby bringing more nodes into an effective load-bearing range, raising the lower portion of the stress distribution, and increasing the overall load level rather than simply eliminating the peak stress. This interpretation is consistent with the macroscopic response: at constant material volume, improved material utilization increases both effective stiffness and offset yield strength.

**Figure 7:**
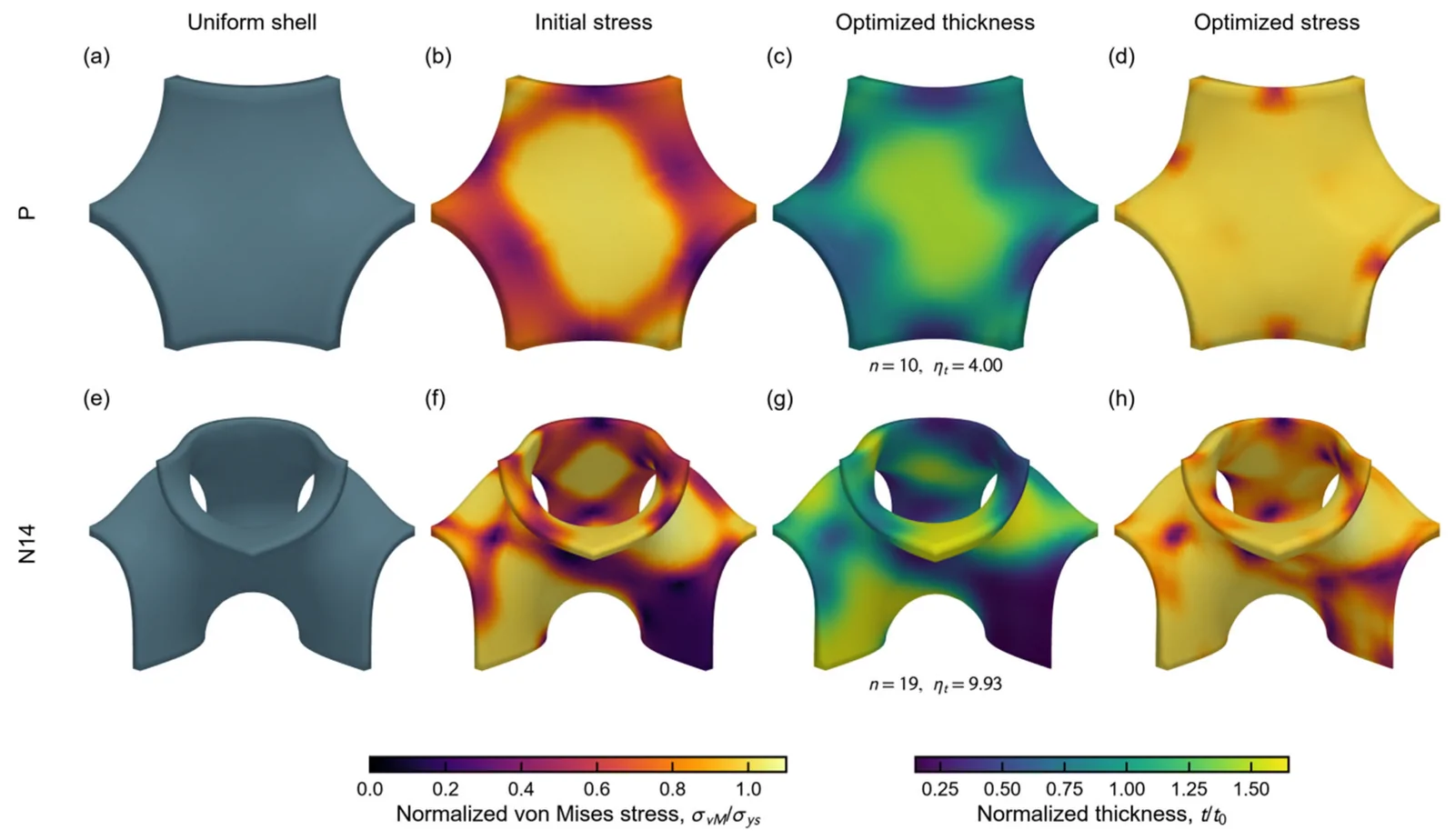
Spatial-field comparison for the P and N14 shell lattices at 10% RD: (**a**,**e**) uniform-thickness shell geometries; (**b**,**f**) normalized von Mises stress of the uniform-thickness baselines at 1% nominal strain; (**c**,**g**) normalized thickness of the representative states under *η*_t_ ≤ 10; and (**d**,**h**) normalized von Mises stress of the corresponding representative states at 1% nominal strain. The representative P and N14 states are *n* = 10, *η*_t_ = 4.00 and *n* = 19, *η*_t_ = 9.93, respectively. Thickness is normalized by the initial uniform thickness *t*_0_, and stress is normalized by the parent-material yield strength *σ*_ys_.

The preceding statistics describe the overall change in the stress distribution. Figure 7 further compares, from a spatial-field perspective, the uniform-thickness geometry, initial stress field, representative thickness field under *η*_t_ ≤ 10, and corresponding stress field for the P and N14 models at 10% RD. In both topologies, the optimized thickened regions generally coincide with highly stressed load-bearing bands in the baseline model, whereas initially low-stress regions are thinned. After reanalysis, a larger portion of the shell reaches an intermediate stress level, and load transfer expands from a few localized regions into more continuous surface paths. The spatial fields confirm that the performance gains arise from reinforcement of load paths and transfer of underutilized material at constant material volume, rather than from an overall increase in wall thickness.

The larger reduction in *CV*_s_ for the P topology than for the N14 topology does not imply superior macroscopic performance. Nodal-stress statistics are affected by topology, node count, and the local plastic state at 1% nominal strain and are appropriate only for comparisons with the baseline on the same topology and mesh. Cross-topology performance also depends on surface geometry, load-path continuity, and structural efficiency in the target direction. The greater stiffness gain of the N14 lattice despite its higher residual stress dispersion indicates that continuous thickness contrast further reinforces favorable load paths, whereas the low- and intermediate-RD P models reach a plateau earlier along the present update trajectory. Thickness redistribution, stress homogenization, and macroscopic performance are therefore clearly related but are not in one-to-one correspondence.

While the S3 shell formulation captures both membrane and bending behaviors, the current thickness-update scheme relies exclusively on mid-surface (SP3) stresses. By omitting outer-surface stresses and bending moments, this mid-surface-driven redistribution strategy is inherently tied to the prescribed loading conditions, necessitating further validation in strongly bending-dominated regions. Future numerical improvements should incorporate through-thickness stress variation into the feedback variable, while also strictly avoiding direct cross-mesh comparisons of nodal stress statistics. Beyond these numerical constraints, physical manufacturability must also be addressed. Because defect mechanisms are highly process-specific—whether in conventional metal forming [44] or laser additive manufacturing—rigorous, process-dependent defect assessments are essential before these numerical designs can be considered fully manufacturable.

### 3.4. Normalized Specific Performance, Scaling Relations, and Design Selection

**Figure 8:**
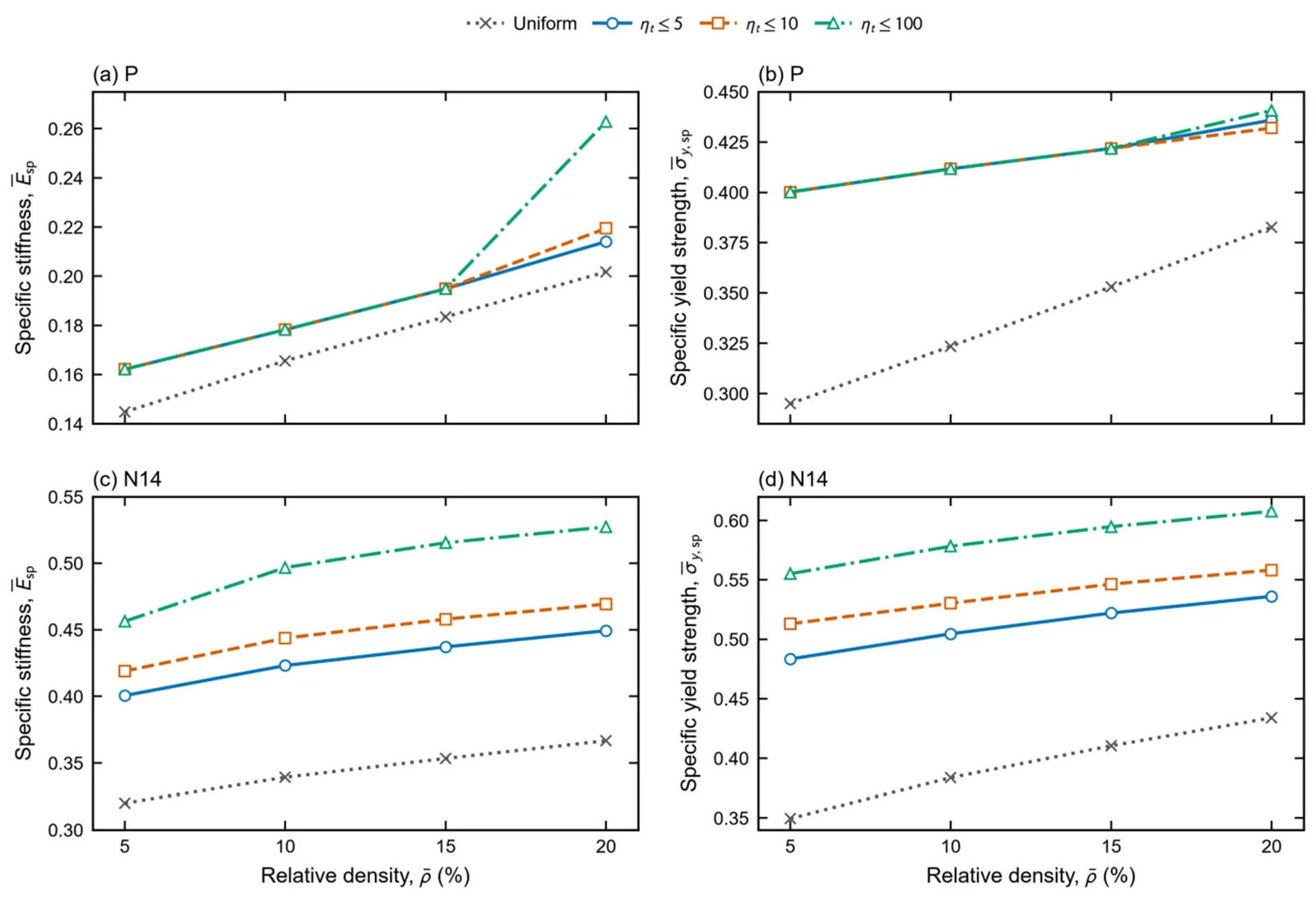
Normalized specific properties of the uniform-thickness baselines and representative states in the three thickness-ratio ranges: (**a**) P specific stiffness; (**b**) P specific yield strength; (**c**) N14 specific stiffness; and (**d**) N14 specific yield strength. Panel-specific vertical limits are used to show the numerical separation among the design ranges without altering any data values.

To remove the influence of the magnitudes of RD and parent-material properties from direct comparisons, the normalized specific stiffness and specific yield strength defined in Equation (9) are used to evaluate the representative states. As shown in Figure 8, the specific properties of both topologies increase gradually with RD, but the gains from thickness redistribution and their sensitivity to the constraint differ markedly. For the P topology, the representative states under *η*_t_ ≤ 5, *η*_t_ ≤ 10, and *η*_t_ ≤ 100 coincide at RD values of 5%, 10%, and 15%; the three nonuniform-thickness curves therefore largely overlap at these densities. Only the 20% model exhibits a pronounced additional increase in specific stiffness within the wide design space. By contrast, the N14 topology improves consistently as the thickness-ratio limit is relaxed at all four RD values, and the spacing between its curves remains relatively uniform.

Within the manufacturing-oriented range *η*_t_ ≤ 5, the normalized specific stiffness and yield strength of the P lattice are 0.162–0.214 and 0.400–0.436, respectively; the corresponding ranges for the N14 lattice are 0.401–0.449 and 0.483–0.536. The N14 lattice therefore provides 2.10–2.47 times the specific stiffness and 1.21–1.24 times the specific yield strength of the P lattice. When the limit is relaxed to *η*_t_ ≤ 10, the N14 ranges increase to 0.419–0.469 and 0.513–0.558, which are 2.14–2.58 and 1.28–1.30 times the corresponding P values. This advantage persists at all four RD values, indicating that, under the present axial loading, material model, and design rule, the N14 topology achieves not only larger absolute improvements but also higher performance per unit material volume.

To characterize sensitivity to RD, a power law is fitted to the four RD points for each group of representative states:

(10) $$\frac{E^{\mathrm{eff}}}{E_{s}}=C_{E}{\bar{\rho }}^{n_{E}},\frac{\sigma_{y}^{\mathrm{eff}}}{\sigma_{\mathrm{ys}}}=C_{\sigma }{\bar{\rho }}^{n_{\sigma }}.$$

Here, *C_E_* and *C_σ_* are the fitted prefactors, whereas *n_E_* and *n_σ_* are the fitted power-law exponents. *n*_E_ − 1 and *n*_σ_ − 1 are the logarithmic slopes of normalized specific stiffness and specific yield strength with respect to RD. The fits in Table 4 summarize only the numerical trends within the 5–20% range studied here and are not intended as universal scaling laws for extrapolation to other densities, unit-cell sizes, or loading conditions.

For the uniform-thickness P lattice, the stiffness exponent *n*_E_ = 1.235 exceeds the N14 value of 1.097, indicating that the specific stiffness of the P topology is more sensitive to RD. After constrained thickness redistribution, *n*_E_ remains greater than 1 for both topologies, consistent with the increase in specific stiffness with density in Figure 8. The change in the yield exponent is more pronounced: the uniform-thickness values of 1.184 and 1.154 for the P and N14 lattices decrease to 1.054–1.074 under the moderate constraints. Nonuniform thickness therefore makes the normalized yield strength approximately linear in RD; equivalently, the normalized specific yield strength becomes nearly independent of RD over the studied range. This behavior is consistent with the increased participation of initially low-stress nodes observed in Section 3.3 and suggests that the feedback design improves yield utilization consistently over the range of material volumes considered.

The coefficient of determination for the P stiffness fit under *η*_t_ ≤ 100 is 0.9844, lower than those of the other groups. This is not a numerical error; rather, the computed trajectories at the four densities do not reach the same degree of thickness differentiation. The 5–15% models cease to improve at *η*_t_ ≈ 4–5, whereas the 20% model extends to *η*_t_ = 49.05 and obtains an additional stiffness gain. The fit therefore combines the effects of RD and the accessible design space along the trajectory, and its exponent should not be used to extrapolate the wide-design-space performance of the P topology. By comparison, all four N14 representative states in the wide design space have actual thickness ratios close to 100, giving a more consistent cross-density trend; nevertheless, their locally ultrathin regions still fail to satisfy the dimensional constraints required for direct engineering implementation.

The results of Section 3.1, Section 3.2, Section 3.3 and Section 3.4 support three levels of design guidance. First, when the objective is to estimate the numerical upper limit of the algorithm at constant material volume, *η*_t_ ≤ 100 reveals a stiffness-improvement potential of approximately 44–46% for the N14 topology and serves as a numerical performance reference. Second, when local dimensions and performance must both be considered, *η*_t_ ≤ 10 provides approximately 28–31% stiffness improvement, 29–47% yield improvement, and the highest specific performance among the moderately constrained N14 designs; it is therefore the preferred window for subsequent specimen development. Finally, when fabrication permits only conservative thickness variation, *η*_t_ ≤ 5 still preserves an approximately 23–25% stiffness gain in the N14 lattice. Low- and intermediate-RD P models also provide stable yield gains at actual thickness ratios of approximately 4–5 and exhibit early saturation of thickness differentiation. For engineering translation, the admissible thickness contrast should be selected jointly with the target cell size, printing resolution, and application-specific dimensional requirements.

### 3.5. Comparison with Existing Methods and Scope Limitations

Previous studies have adopted different strategies for designing variable-thickness TPMS shell lattices. Fan et al. [28] and Rizza et al. [29] used prescribed thickness gradients, while Ma et al. [30] employed strain-energy sensitivities to improve elastic isotropy. Zhang et al. [31] and Jia et al. [32] introduced local reinforcement according to the stress distribution of a uniform-thickness structure. These methods have demonstrated the benefits of non-uniform thickness, but their thickness patterns are generally determined by predefined functions, regional classifications, or the initial mechanical response. In contrast, the present method updates the nodal thickness continuously according to the stress field recalculated in each iteration. The material distribution can therefore evolve as the load-transfer path changes, without requiring predefined reinforcement regions or discrete thickness levels. Spatial filtering is used to reduce abrupt thickness variations, and volume rescaling maintains the prescribed material volume after each update. This equal-volume condition makes it possible to attribute the improvement to material redistribution rather than additional material. The method also uses standard finite-element stress outputs and does not require the explicit derivation of response sensitivities. The same procedure was applied to both P and N14 structures over relative densities of 5–20%, indicating that the framework is not restricted to a single topology or density. Moreover, designs satisfying different thickness-ratio limits can be selected from the same iterative trajectory, providing a convenient way to balance mechanical performance against the degree of thickness variation.

Under *η_t_* ≤ 10, the present P designs achieved stiffness improvements of 6.20–12.00% and yield-strength improvements of 12.88–35.64% over RDs of 5–20%. Zhang et al. [31] reported elastic-modulus increases of 17.0–23.5%, depending on the evaluation procedure, a density-normalized modulus increase of 9.2%, and a yield-strength increase of 10.2%. Jia et al. [32] reported increases of 1.9% and 10.6% in relative elastic modulus and 6.5% and 15.4% in relative ultimate strength for two locally reinforced P designs. These results indicate that the stiffness improvement of the present P designs is of a similar order to the density-normalized results reported previously, but does not consistently exceed all existing P-type designs. The improvement in yield strength is more pronounced, particularly at lower RDs. However, the strength results from Ref. [32] are not directly equivalent to the present results because ultimate strength and 0.2% offset yield strength represent different stages of the compressive response.

Under *η_t_* ≤ 10, the stiffness and yield strength of N14 increased by 27.96–30.90% and 28.64–46.75%, respectively. These gains are considerably greater than those obtained for the P structures using the same design procedure. This result suggests that N14 provides more favorable load paths for stress-driven material redistribution and is more responsive to continuous thickness optimization. It should not, however, be interpreted as direct evidence that the present method outperforms Refs. [31,32], since the difference is partly associated with the underlying topology. Instead, the comparison between P and N14 demonstrates that the effectiveness of variable-thickness design is topology-dependent.

A key limitation of this study is that all optimized thickness fields were derived exclusively from uniaxial compression along a single selected axis at 1% nominal strain. Consequently, the resulting thickness redistribution is inherently load-path-specific and should not be construed as evidence of isotropic or generalized multiaxial performance enhancement. Specifically, material removed from regions lightly loaded under the present uniaxial condition might otherwise contribute to stiffness or strength under shear, off-axis compression, or combined loading modes—responses that were not evaluated here and could potentially degrade. To address this, future multi-load optimization formulations should integrate axial, shear, and off-axis cases via weighted or worst-case objective functions, enforce minimum-performance constraints across all load cases, and rigorously validate the resulting thickness fields under loading modes not involved in the optimization update. Until such comprehensive evaluations are completed, the reported mechanical gains remain strictly restricted to the prescribed axial compression path.

### 3.6. Algorithmic-Parameter and Mesh-Sensitivity Analysis Results

**Figure 9:**
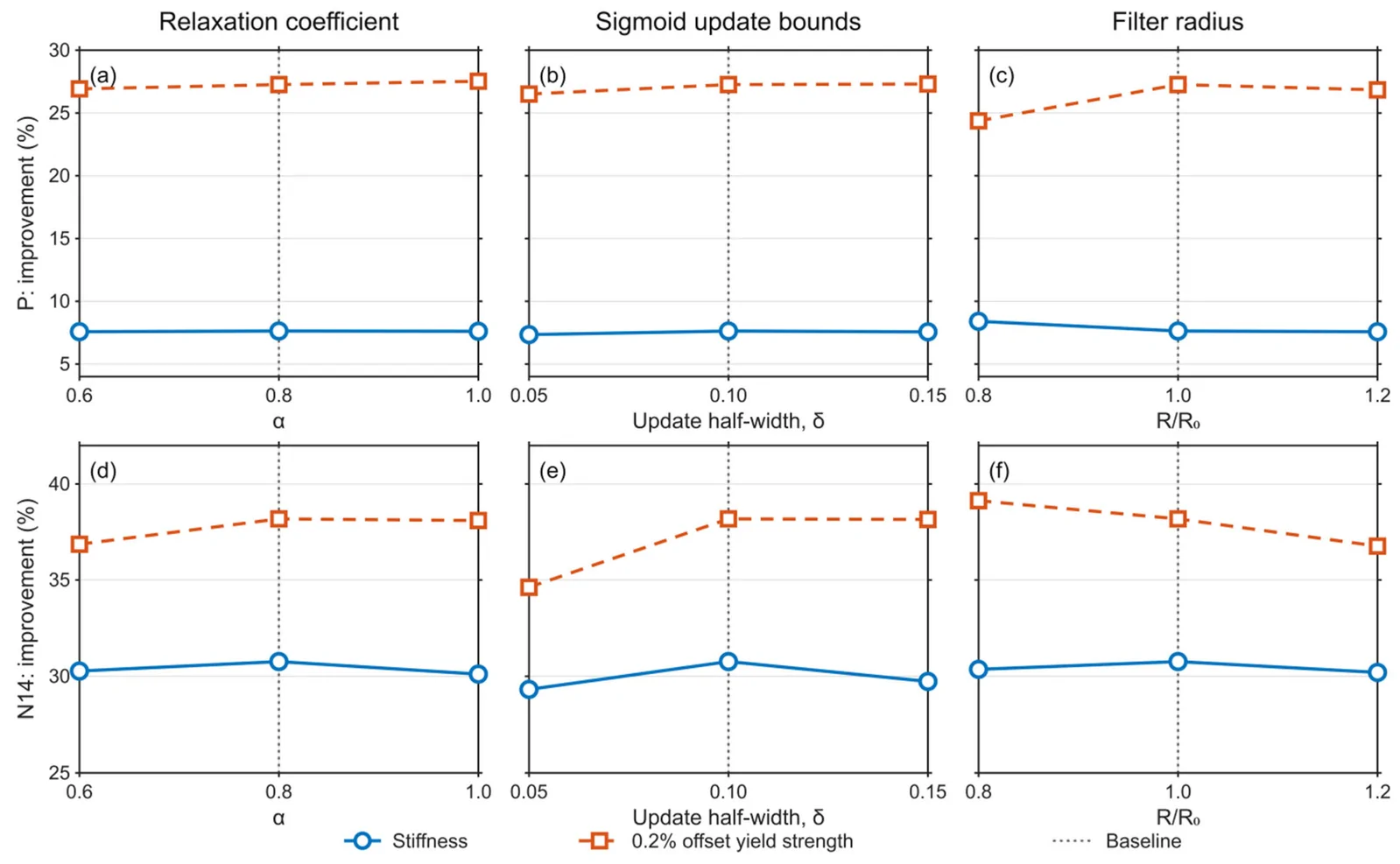
Sensitivity of stiffness and 0.2% offset yield-strength improvements to (**a**,**d**) the relaxation coefficient, (**b**,**e**) the sigmoid-based update bounds, and (**c**,**f**) the normalized filter radius for the P and N14 topologies at an RD of 10%. The dotted vertical lines denote the baseline parameter settings. Each point represents the state with the highest effective Young’s modulus among the numerically sampled states satisfying a thickness-ratio limit of 10.

Figure 9 summarizes the performance of the representative states satisfying a thickness-ratio limit of 10 after varying the relaxation coefficient, sigmoid update bounds, and filter radius. For P, the stiffness-gain ranges are 7.58–7.63%, 7.35–7.63%, and 7.57–8.40% for the three parameter families, respectively; the corresponding yield-strength-gain ranges are 26.92–27.53%, 26.51–27.31%, and 24.37–27.27%. For N14, the corresponding stiffness-gain ranges are 30.11–30.76%, 29.32–30.76%, and 30.20–30.76%, while the yield-strength-gain ranges are 36.86–38.18%, 34.62–38.18%, and 36.76–39.13%.

The relaxation coefficient mainly changes the rate at which thickness differentiation develops: smaller α values require more iterations, whereas the attainable improvements remain similar. Narrower sigmoid bounds likewise produce more conservative updates and longer trajectories. The filter radius has a greater influence on the local thickness distribution and the yield response. In contrast, the filter radius has a more pronounced influence on the local thickness distribution and yield response. For the P topology, reducing the filter radius to 0.8*R*_0_ produces the highest stiffness gain but a lower yield-strength gain, indicating that a thickness distribution favorable for initial axial stiffness does not necessarily alleviate the critical stress concentration governing first yield. For the N14 topology, decreasing the filter radius from *R*_0_ to 0.8*R*_0_ increases the yield-strength gain from 38.18% to 39.13%, whereas the stiffness gain decreases slightly from 30.76% to 30.20%. This result suggests that weaker spatial smoothing preserves more localized thickness variations, which may more effectively redistribute material around the critical stress concentrations controlling initial yield. However, the present results do not demonstrate that further reductions in the filter radius would continuously improve yield strength or establish a higher theoretical performance bound. An excessively small filter radius may produce node-to-node thickness oscillations, mesh-dependent patterns, and abrupt local thickness gradients that are less compatible with subsequent manufacturing-oriented design. The baseline radius *R*_0_ was therefore retained for the main analysis because its filtering neighborhood spans at least the local adjacent-node distance, providing a compromise between local material-redistribution freedom and a smooth, mesh-robust thickness field.

The three termination thresholds do not change the selected states for the N14 trajectories and affect only a subset of the P trajectories. The largest threshold-induced differences are 0.76 percentage points in stiffness gain and 2.54 percentage points in yield-strength gain, both for P with a filter radius of 0.8 times the baseline value. All examined parameter combinations nevertheless retain positive stiffness and yield-strength gains, and the larger improvement of N14 relative to P remains unchanged. The principal stiffness trends are therefore robust within the examined intervals, while the precise yield-strength values and local thickness fields exhibit moderate parameter and trajectory dependence.

**Figure 10:**
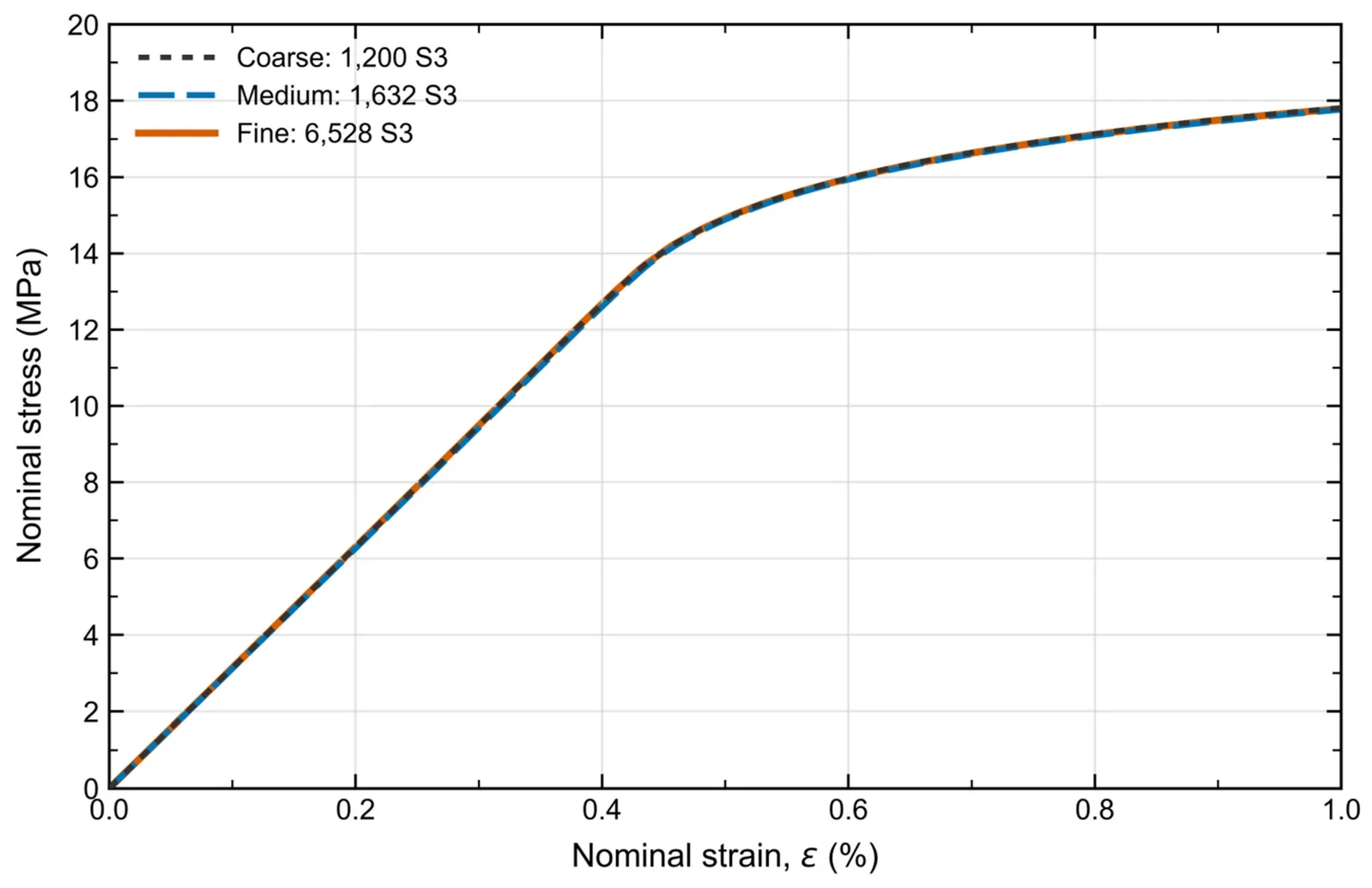
Nominal stress–strain responses of the uniform-thickness P lattice at 10% RD for the coarse, medium, and fine surface discretizations.

Figure 10 compares the nominal stress–strain curves obtained from the three P-lattice surface discretizations at 10% RD. The curves nearly overlap throughout the prescribed loading range. The initial linear fits also remain consistent, with coefficients of determination greater than 0.999999 for all three meshes. As shown in Table 5, relative to the 6528-element mesh, the maximum absolute deviations among the two coarser meshes are 0.608% for the effective Young’s modulus, 0.129% for the 0.2% offset yield strength, and 0.131% for the nominal stress at 1% strain. The small discrepancies and the nearly coincident curves indicate that the original 1632-element P discretization is sufficiently insensitive to further refinement for the global quantities considered under the prescribed loading condition. The mesh convergence test results for the N14 model show similar trends.

## 4. Conclusions

A stress-feedback-driven continuous variable-thickness method was developed to improve the axial stiffness and yield strength of P and N14 TPMS shell lattices without changing their mid-surface topology or material volume. The method redistributes material from lightly loaded regions to the main load-transfer paths through repeated stress-based thickness updates, spatial filtering, and volume normalization. Over RDs of 5–20%, the stiffness of N14 increased by 42.55–46.39% in the wide numerical design space. When the thickness ratio was limited to ηt ≤ 10, N14 retained stiffness improvements of 27.96–30.90% and yield-strength improvements of 28.64–46.75%. The response of P was more dependent on RD, with the 20% RD model reaching a stiffness improvement of 30.28% in the wide design space. The reduction in nodal-stress dispersion further indicates that the improvement resulted from more effective material utilization. The different responses of P and N14 also show that the effectiveness of variable-thickness design depends strongly on the underlying topology.

The parameter study at 10% RD showed that the main stiffness trends were maintained under the examined variations in the relaxation coefficient, sigmoid update bounds, filter radius, and termination criterion. The yield-strength results were somewhat more sensitive to the filter radius and update bounds, but all tested parameter combinations produced positive stiffness and strength improvements. The mesh-sensitivity analysis also showed good agreement among the three P discretizations. Relative to the finest mesh, the maximum deviations were 0.608% for the effective Young’s modulus, 0.129% for the 0.2% offset yield strength, and 0.131% for the nominal stress at 1% strain. These results support the use of the baseline discretization for evaluating the global mechanical quantities considered in this study.

The designs satisfying ηt ≤ 5 and 10 provide moderate-contrast candidates that retain substantial performance gains while avoiding the extreme local thickness variations found in the wide design space. However, these thickness-ratio limits do not by themselves demonstrate manufacturability. The present conclusions are based on idealized finite-element models under uniaxial compression along one direction and do not account for manufacturing defects, material variability, absolute minimum wall thickness, build orientation, or curvature-dependent dimensional errors. Future work should incorporate process-specific manufacturing constraints and multiple loading conditions, including shear and off-axis compression. Experimental studies on printed multi-cell specimens are also required to validate the predicted improvements and, where necessary, to calibrate material properties and geometric imperfections through inverse identification. Furthermore, the incorporation of through-thickness stress variation into the feedback variable will be explored in a separate future study to enlarge the design space and seek further enhancement in the stiffness and strength of TPMS shell lattices.

## Figures and Tables

**Table 1:** Representative states selected within the wide design space (*η*_t_ ≤ 100).

Type	RD (%)	*E*_0_*^eff^* (MPa)	n*	η_t_*	E_n__*_^eff^ (MPa)	Enhancement (%)
P	5	1370.3	13	4.97	1534.8	12.00
P	10	3135.0	10	4.00	3374.3	7.63
P	15	5209.9	10	4.04	5533.2	6.20
P	20	7638.3	95	49.05	9951.3	30.28
N14	5	3029.4	42	91.37	4318.5	42.55
N14	10	6422.8	57	94.56	9402.5	46.39
N14	15	10,034.6	47	97.35	14,635.0	45.85
N14	20	13,882.5	48	97.58	19,965.0	43.81

**Table 2:** Representative states and performance improvements under *η*_t_ ≤ 5 and *η*_t_ ≤ 10.

Type	RD (%)	*η_t,_*_5_*	*ΔE*_5_ (%)	*Δσ_y,_*_5_ (%)	*η_t,_*_10_*	*ΔE*_10_ (%)	*Δσ_y,_*_10_ (%)
P	5	4.97	12.00	35.64	4.97	12.00	35.64
P	10	4.00	7.63	27.27	4.00	7.63	27.27
P	15	4.04	6.20	19.48	4.04	6.20	19.48
P	20	4.99	6.11	13.91	9.67	8.79	12.88
N14	5	4.92	25.16	38.29	9.34	30.90	46.75
N14	10	4.92	24.71	31.49	9.93	30.76	38.18
N14	15	4.93	23.69	27.18	9.74	29.57	33.12
N14	20	4.93	22.52	23.53	9.80	27.96	28.64

**Table 3:** Ranges of thickness-redistribution and stress-statistical metrics under moderate thickness-ratio constraints.

Type	Limit	CV_t_*	*r_t,s_*_0_	*CV_s_* Decrease (%)	*s*_95_ Change (%)
P	*η_t_* ≤ 5	0.289–0.379	0.820–0.949	74.31–77.51	−1.26 to −0.89
P	*η_t_* ≤ 10	0.289–0.416	0.668–0.949	74.31–79.36	−1.26 to −0.89
N14	*η_t_* ≤ 5	0.338–0.366	0.918–0.929	37.17–40.30	−0.48 to +0.05
N14	*η_t_* ≤ 10	0.412–0.444	0.882–0.903	44.27–47.31	−0.42 to +0.01

**Table 4:** Power-law exponents for RD scaling under different thickness-design ranges.

Type	State	*n_E_*	*R_E_* ^2^	*n_σ_*	*R_σ_* ^2^
P	Uniform thickness	1.235	0.9995	1.184	0.9993
P	*η_t_* ≤ 5	1.194	0.9990	1.059	0.9998
P	*η_t_* ≤ 10	1.208	0.9981	1.054	0.9999
P	*η_t_* ≤ 100	1.306	0.9844	1.065	0.9996
N14	Uniform thickness	1.097	0.9999	1.154	0.9999
N14	*η_t_* ≤ 5	1.082	1.0000	1.074	1.0000
N14	*η_t_* ≤ 10	1.081	1.0000	1.061	1.0000
N14	*η_t_* ≤ 100	1.105	0.9999	1.065	1.0000

**Table 5:** Representative P-lattice mesh-sensitivity results at 10% RD. Absolute deviations are calculated relative to the fine mesh.

Mesh	Nodes/S3 Elements	E^eff^ (MPa)	ΔE vs. Fine (%)	σ_y_, _0.2%_ (MPa)	Δσ_y_ vs. fine (%)	σ_1%_ (MPa)	Δσ_1%_ vs. Fine (%)
Coarse	661/1200	3136.83	0.041	16.8395	0.129	17.8134	0.121
Medium	865/1632	3119.05	0.608	16.8033	0.086	17.7687	0.131
Fine	3361/6528	3138.12	0.000	16.8178	0.000	17.7919	0.000

## Data Availability

The original contributions presented in this study are included in the article. Further inquiries can be directed to the corresponding author.
